# An interplay of non-coding RNAs regulates *CDH13* expression and affects endothelial function and coronary artery disease risk

**DOI:** 10.21203/rs.3.rs-7333062/v1

**Published:** 2025-08-20

**Authors:** Shuangyue Li, Xiaoning Song, Anastasiia Diagel, Ling Li, Aldo Moggio, Yifan Chen, Zhaolong Li, Tan Dang, Miaomiao Li, Rui Shen, Angela Ma, Marius Schwab, Nicolas Barbera, Constanze Lehertshuber, Amos Romer, Luigi Filippo Brizzi, Johannes Krefting, Nils Krüger, Hendrik Sager, Reinier Boon, Mete Civelek, Casey Romanoski, Aldons Lusis, Andreas Schober, Thorsten Kessler, Moritz von Scheidt, Johan Björkegren, Lars Maegdefessel, Maliheh Nazari-Jahantigh, Heribert Schunkert, Zhifen Chen

**Affiliations:** Department of Cardiology, German Heart Center, TUM University Hospital, TUM School of Medicine and Health, Technical University Munich Deutsches Zentrum für Herz- und Kreislaufforschung (DZHK), partner site Munich Heart Alliance (MHA), Munich, Germany; Department of Cardiology, German Heart Center, TUM University Hospital, TUM School of Medicine and Health, Technical University Munich Deutsches Zentrum für Herz- und Kreislaufforschung (DZHK), partner site Munich Heart Alliance (MHA), Munich, Germany; Department of Cardiology, German Heart Center, TUM University Hospital, TUM School of Medicine and Health, Technical University Munich Deutsches Zentrum für Herz- und Kreislaufforschung (DZHK), partner site Munich Heart Alliance (MHA), Munich, Germany; Department of Cardiology, German Heart Center, TUM University Hospital, TUM School of Medicine and Health, Technical University Munich Deutsches Zentrum für Herz- und Kreislaufforschung (DZHK), partner site Munich Heart Alliance (MHA), Munich, Germany TUM School of Computation, Information and Technology (CIT), Technical University Munich, 85748 Garching bei München, Germany; Department of Cardiology, German Heart Center, TUM University Hospital, TUM School of Medicine and Health, Technical University Munich; Department of Cardiology, German Heart Center, TUM University Hospital, TUM School of Medicine and Health, Technical University Munich Deutsches Zentrum für Herz- und Kreislaufforschung (DZHK), partner site Munich Heart Alliance (MHA), Munich, Germany; Molecular Vascular Medicine, Klinikum rechts der Isar - Technical University Munich Deutsches Zentrum für Herz- und Kreislaufforschung (DZHK), partner site Munich Heart Alliance (MHA), Munich, Germany; Department of Cardiology, German Heart Center, TUM University Hospital, TUM School of Medicine and Health, Technical University Munich Deutsches Zentrum für Herz- und Kreislaufforschung (DZHK), partner site Munich Heart Alliance (MHA), Munich, Germany; Department of Cardiology, German Heart Center, TUM University Hospital, TUM School of Medicine and Health, Technical University Munich Deutsches Zentrum für Herz- und Kreislaufforschung (DZHK), partner site Munich Heart Alliance (MHA), Munich, Germany; Department of Cardiology, German Heart Center, TUM University Hospital, TUM School of Medicine and Health, Technical University Munich Deutsches Zentrum für Herz- und Kreislaufforschung (DZHK), partner site Munich Heart Alliance (MHA), Munich, Germany; Department of Genetics and Genomic Sciences, Institute of Genomics and Multiscale Biology, Icahn School of Medicine at Mount Sinai; Department of Cardiology, German Heart Center, TUM University Hospital, TUM School of Medicine and Health, Technical University Munich; Center for Public Health Genomics, University of Virginia; Department of Cardiology, German Heart Center, TUM University Hospital, TUM School of Medicine and Health, Technical University Munich Deutsches Zentrum für Herz- und Kreislaufforschung (DZHK), partner site Munich Heart Alliance (MHA), Munich, Germany; Department of Cardiology, German Heart Center, TUM University Hospital, TUM School of Medicine and Health, Technical University Munich Deutsches Zentrum für Herz- und Kreislaufforschung (DZHK), partner site Munich Heart Alliance (MHA), Munich, Germany; Department of Cardiac-Thoracic-Vascular Sciences and Public Health, University of Padova, Padova, Italy.; Department of Cardiology, German Heart Center, TUM University Hospital, TUM School of Medicine and Health, Technical University Munich Deutsches Zentrum für Herz- und Kreislaufforschung (DZHK), partner site Munich Heart Alliance (MHA), Munich, Germany; Department of Cardiology, German Heart Center, TUM University Hospital, TUM School of Medicine and Health, Technical University Munich Deutsches Zentrum für Herz- und Kreislaufforschung (DZHK), partner site Munich Heart Alliance (MHA), Munich, Germany; Department of Cardiology, German Heart Center, TUM University Hospital, TUM School of Medicine and Health, Technical University Munich Deutsches Zentrum für Herz- und Kreislaufforschung (DZHK), partner site Munich Heart Alliance (MHA), Munich, Germany; Goethe University Hospital, Institute for Cardiovascular Regeneration; Center for Public Health Genomics, University of Virginia Department of Biomedical Engineering, University of Virginia; Department of Cellular and Molecular Medicine, The University of Arizona; David Geffen School of Medicine, University of California; Institute for Cardiovascular Prevention (IPEK), Ludwig-Maximilians-Universität München Deutsches Zentrum für Herz- und Kreislaufforschung (DZHK), partner site Munich Heart Alliance (MHA), Munich, Germany; Department of Cardiology, German Heart Center, TUM University Hospital, TUM School of Medicine and Health, Technical University Munich Deutsches Zentrum für Herz- und Kreislaufforschung (DZHK), partner site Munich Heart Alliance (MHA), Munich, Germany; Department of Cardiology, German Heart Center, TUM University Hospital, TUM School of Medicine and Health, Technical University Munich Deutsches Zentrum für Herz- und Kreislaufforschung (DZHK), partner site Munich Heart Alliance (MHA), Munich, Germany; Department of Medicine, Huddinge, Karolinska Institutet, Karolinska Universitetssjukhuset Department of Genetics and Genomic Sciences, Institute of Genomics and Multiscale Biology, Icahn School of Medicine at Mount Sinai; Molecular Vascular Medicine, Klinikum rechts der Isar - Technical University Munich Deutsches Zentrum für Herz- und Kreislaufforschung (DZHK), partner site Munich Heart Alliance (MHA), Munich, Germany; Institute for Cardiovascular Prevention (IPEK), Ludwig-Maximilians-Universität München Deutsches Zentrum für Herz- und Kreislaufforschung (DZHK), partner site Munich Heart Alliance (MHA), Munich, Germany; Department of Cardiology, German Heart Center, TUM University Hospital, TUM School of Medicine and Health, Technical University Munich Deutsches Zentrum für Herz- und Kreislaufforschung (DZHK), partner site Munich Heart Alliance (MHA), Munich, Germany; Department of Cardiology, German Heart Center, TUM University Hospital, TUM School of Medicine and Health, Technical University Munich Deutsches Zentrum für Herz- und Kreislaufforschung (DZHK), partner site Munich Heart Alliance (MHA), Munich, Germany

**Keywords:** Coronary artery disease (CAD), long non-coding RNA (lncRNA), microRNA (miRNA), GWAS-eQTL colocalization analysis, dRfxCas13d, CRISPR/Cas9, CRISPR activation (CRISPRa), 3’ untranslated region (3’ UTR), RNA stability, RNA interference

## Abstract

Many common diseases have a polygenic architecture. The responsible alleles are thought to mediate risk by disturbing gene regulation in most cases, however, the precise mechanisms have been elucidated only for a few. Here, we investigated the *16q23.3* genomic locus, which genome-wide significantly associates with coronary artery disease, a globally leading cause of death caused by accumulation of lipid-rich inflammatory plaques in the arterial wall. The locus harbors *CDH13*, whose mRNA and protein we found to be suppressed in atherosclerotic human and mouse arteries. Loss-of-function(LoF) variants of *CDH13* were associated with detrimental cardiovascular phenotypes in the UK Biobank. Its knock-out increased plaque-sizes in *Cdh13*^*−/−*^/*Apoe*^*−/−*^ mice compared to *Apoe*^*−/−*^ mice on a Western diet. After establishing an atheroprotective role of CDH13, we studied its regulation. Integration of population genomic and transcriptomic datasets by GWAS-eQTL colocalization analysis identified *CDH13* and four long non-coding RNAs (lncRNAs) as candidate causal genes at the *16q23.3* locus. dCas13-mediated RNA immunoprecipitation revealed that the lncRNA *CDH13-AS2* binds to *CDH13* mRNA in human endothelial cells (ECs). Its CRISPR/Cas9-based knockout in ECs was atherogenic, whereas dCas9-based transcriptional activation (CRISPRa) of *CDH13-AS2* was atheroprotective; effects that were found to be mediated by the stability of *CDH13* mRNA. To further understand how the *CDH13-AS2* protects the mRNA we searched *in silico* and screened *in vitro* for microRNAs (miRNAs) that bind to *CDH13* 3’UTR. Indeed, four miRNAs, miR-19b-3p, miR-125b-2–3p, miR-433–3p, and miR-7b-5p, were found experimentally to accelerate *CDH13* mRNA degradation, an effect that was neutralized by CRISPRa of *CDH13-AS2*. Taken together, our study demonstrates an interplay of miRNAs, lncRNAs, and mRNA, which modulates the abundance of an atheroprotective protein in endothelial cells, which may offer a new therapeutic target for coronary artery disease.

## Introduction

Coronary artery disease (CAD) is a genetically-mediated and often devastating common disease. Genome-wide association studies (GWAS) identified hundreds of risk alleles that cumulatively shape a population-wide disposition for atherosclerosis^1–3^, i.e. the build-up of lipid-rich inflammatory plaques in arterial vessels, which ultimately may occlude and cause myocardial infarction. CAD is also a prototypic polygenic disease that is primarily precipitated by disturbed gene expression rather than by structural changes of the affected proteins^1, 4, 5^. However, the molecular mechanisms that link CAD risk alleles with altered gene expression have been elaborated in only a few exceptional cases^6–8^.

**In human biology, a large fraction of** key regulators of **tissue-specific gene expression are** non-coding RNAs (ncRNAs), such as long non-coding RNA (lncRNA) and microRNA (miRNA). Such RNAs regulate the expression, splicing, stability, or translation of the cognate protein-coding genes either by interacting directly with the mRNA or indirectly via binding to other ncRNAs^9–14^. Therapeutically, the modulating effects of ncRNAs can be harnessed by either silencing or activating genes^15, 16^. For instance, a phase 2 clinical trial showed that inhibition of miR-132 by an antisense oligonucleotide may prevent cardiac remodeling post-myocardial infarction^17^. Several lncRNA coding genes mapped to CAD-GWAS loci^3^, and the most significant CAD locus is mapped to the lncRNA coding gene, *ANRIL*^16^.

Since 2017, the *16q23.3* locus has been associated with CAD by GWAS and repeatedly replicated by studies with ever-growing sample sizes and ethnic diversity^1, 2, 18 19^. Yet, the functional implications of this locus remains unclear.

Our exploration revealed that the *16q23.3* locus, residing in the *CDH13*(cadherin 13, T cadherin) regulates both the protein-coding and lncRNA-coding genes. Both our mouse and human data indicated that genetic loss of *CDH13* is atherogenic, which aligned with the observation that lower *CDH13* expression was found in arterial tissue from patients and mice with atherosclerosis. In a search for potential therapeutic RNA targets, we experimentally identified a lncRNA and four miRNAs to participate in the regulation of *CDH13* mRNA. A series of CRISPR/Cas9-based knockdown and dCas9-based transcriptional activation (CRISPRa) experiments in human endothelial cells clarified the role of interacting ncRNAs in regulating *CDH13* mRNA and consolidated the notion of an atheroprotective role of *CDH13*.

## Results

### Five candidate causal genes are identified at the *16q23.3* locus

**Figure 1:**
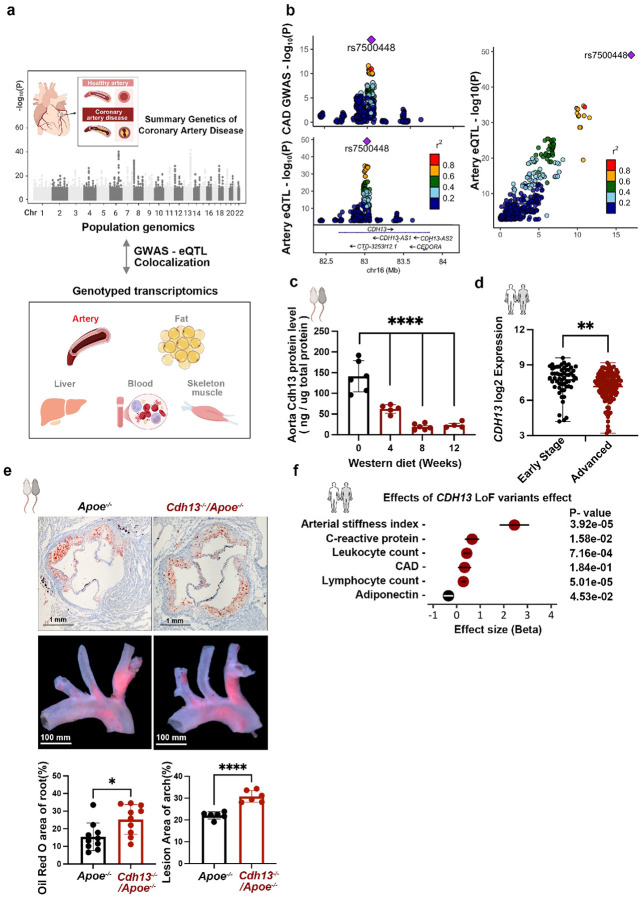
Prioritization of five candidate genes at the *16q23.3* locus. **a,** The scheme of colocalization analysis of GWAS and tissue eQTL. For the GWAS, we used the latest coronary artery disease summary genetic statistics of CARDIoGRAMplusC4D Consortium^1^. For the eQTLs, five types of genotyped transcriptome from ~ 600 individuals were used, **b**, eQTL-GWAS colocalization analysis suggested *CDH13* and four IncRNAs to be candidate genes of CAD in the artery (method, supplement Tablel). **c**, Cdh13 protein in aorta was reduced in mice on weeks of Western diet (ordinary one-way ANOVA multiple comparisons, ****, P ≤ 0.0001). **d**, Artery expression of *CDH13* decreased in advanced carotid atherosclerosis plaques (n=145) compared to early-stage plaques (n=57) (supplement Table 2, multiple unpaired t-tests, **, P ≤ 0.01). **e**, Atherosclerosis lesion quantification in aortic roots and arch of the *Cdh13*^*−/−*^*/Apoe*^−/−^ and the *Apoe*^*−/−*^ mice after eight weeks of Western diet feeding. The quantification was the lesion area dividing the total artey tissue area. Unpaired t-test, *, P ≤ 0.05; ****, P ≤ 0.0001. **f.** Loss of function (LoF) variants of *CDH13* associated with CAD and detrimental cardiovascular phenotypes in UK biobank cohort. Phenotypes with P < 0.5 are shown.(method, supplement Table 3 and 4).

The CAD association signals at the *16q23.3* locus reside within the intragenic region of the protein-coding gene, *CDH13* (cadherin 13, T cadherin). To systematically map candidate causal genes at this locus, we conducted a colocalization analysis using the CAD GWAS signal at the *16q23.3* locus and expression quantitative trait locus (eQTL) datasets from disease-relevant tissues (Method, Figure.1a and 1b). The GWAS association signal was from the latest summary genetic statistics of the CARDIoGRAMplusC4D Consortium^1^. The tissue types explored for eQTL analyses included arteries, adipose tissues, liver, blood, and skeletal muscle of ~ 600 individuals from the Stockholm-Tartu Atherosclerosis Reverse Networks Engineering Task (STARNET) project^20, 21^ (Method, Figure.1a). The results colocalized the CAD GWAS signal at the *16q23.3* locus with an eQTL signal specific to arterial tissues pointing to *CDH13* and four lncRNAs (*CDH13-AS1*, *CDH13-AS2*, *CEDORA*, and *CTD-3253I12.1*) to be candidate causal genes for CAD (Method, Figure. 1b, Extended Data Figure 1a, supplementary Table 1). Namely, their expressions in arterial tissues likely mitigated the risks of CAD diseases.

### The loss of *CDH13* promotes the development of atherosclerosis in humans and mice.

To understand the underlying mechanism of CAD related to the *16q23.3* locus, a critical step is to elucidate the functionality of the protein-coding gene, *CDH13*. We first analyzed the animal model for atherosclerosis, in the *Apoe*^*−/−*^ mouse, theCdh13 protein levels gradually declined in the aorta after 4, 8, and 12 weeks of Western diet treatment (Figure. 1c, Extended Data Figure 1b). Likewise, the bulk RNA sequencing of atherosclerosis plaques from patients undergoing carotid endarterectomy (Munich Vascular Biobank)^22, 23^ revealed in advanced plaques (n=145) less *CDH13* expression than in early plaques (n=57) (Method, Figure. 1d, supplementary Table 2). Thus, we observed reduced arterial expression of *CDH13* during the development of atherosclerosis in both humans and mice, suggesting a protective role of CDH13. To confirm this hypothesis, we generated *Cdh13*^*−/−*^ gene knockout (KO) mice on the atherogenic background by crossing *Cdh13*^*−/−*^ with *Apoe*^*−/−*^ mice to obtain the *Cdh13*^*−/−*^/*Apoe*^*−/−*^ mice(Extended Data Figure 1c and 1d). Compared to the *Apoe*^*−/−*^ mice, the *Cdh13*^*−/−*^/*Apoe*^*−/−*^ mice on an eightweek Western diet (WD)(Extended Data Figure 1e) had increased atherosclerosis lesion areas in the aortic root and arch (Method, Figure. 1e, Extended Data Figure 1f), reinforcing *CDH13* to be atheroprotective. To further assess the roles of *CDH13*, we investigated the effect of its loss-of-function (LoF) variants on CAD and other 19 vascular-related diseases or traits using phenotype and exome sequencing data from the 470,000 UK Biobank participants (Method, Supplementary Table 3 and 4). LoF variants of *CDH13* were found in 272 participants who showed a trend of increased CAD incidence (β = 0.335, P = 0.184). LoF variants were significantly associated with atherogenic traits, such as increased arterial stiffness index (β = 2.450, P=3.92e-5), serum C-reactive protein level (β = 0.654, P=1.58e-2), blood leukocyte and lymphocyte counts (β=0.427 and 0.281, P=7.16e-4 and 5.01e-5, respectively), but decreased serum adiponectin level (β = −0.349, P=4.53e-2) (Figure 1f, Supplementary Table 3 and 4). Together, the results from mice and humans suggest that *CDH13* is a causal gene at the *16q23.3* locus (Figure 1), with higher expression appearing to be protective against CAD.

### All five candidate causal genes are expressed in human endothelial cells

**Figure.2:**
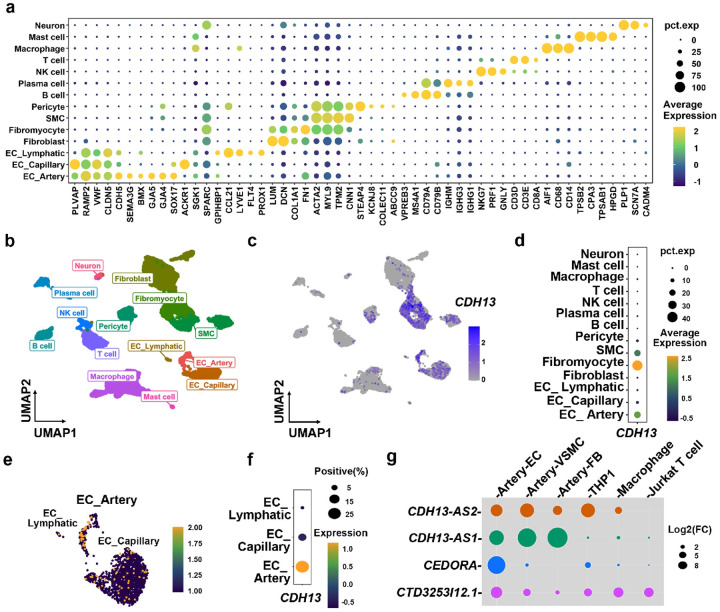
LncRNAs and *CDH13* at *16q23.3* locus highly expressed in endothelial cells. **a,** Cell markers for clustering the single-cell RNA-seq dataset of human coronary artery **(b** through **f). b,** Mapped cell clusters of the human coronary artery single-cell RNAseq dataset. **c** and **d,** The relative expression level of *CDH13* per cell type, **e** and **f,** The relative expression level of *CDH13* in endothelial (EC) sub-populations(method). **g,** Expression patterns of the four lncRNAs in cell types of the vascular wall, including human coronary artery endothelial cells(ECs), human artery smooth muscle cells (VSMCs), human artery fibroblasts (FBs), THP1 monocytes, THP1 macrophages, and Jurkat T cells. The lncRNA expression level was first normalised to *GAPDH* and then compared with HEK.293T cells. The bubble size indicated the log2(fold of change, FC, N = 5)(Extended Data Figure 2).

Beyond *CDH13*, our eQTL analysis also prioritized four lncRNAs as candidate causal genes at the *16q23.3* locus (Figure. 1b). To identify relevant cell type(s) that express the respective RNAs we analyzed the publicly available single-cell (sc) RNA-seq dataset of proximal-to-mid right coronary artery (RCA) from four patients who underwent heart transplantation(Methods)^24^. Our analysis identified 14 major cell populations in this dataset(Method, Figure. 2a and 2b), and *CDH13* was highly expressed in vascular muscle cells (VSMCs), fibromyocytes, and endothelial cells (ECs), especially arterial ECs (Method, Figure. 2c to 2f). Unfortunately, none of the four lncRNAs were identified in the scRNAseq datasets due to the low-expression nature of lncRNAs. Thus, we did qPCR experiments for the four lncRNAs in cardiovascular cell types, including human coronary artery ECs, VSMCs, fibroblasts (FBs), monocytes (the THP1 cell line), macrophages (THP1-differentiated), and T cells (the Jurkat cell line). As a result, EC was the only cell type expressing all four lncRNAs with a relatively high level (Figure. 2g, Extended Data Figure 2a to 2d). Therefore, our subsequent investigations focused on ECs to study the interaction between lncRNA and *CDH13*.

### LncRNA interacts with *CDH13* mRNA in human endothelial cells

**Figure.3:**
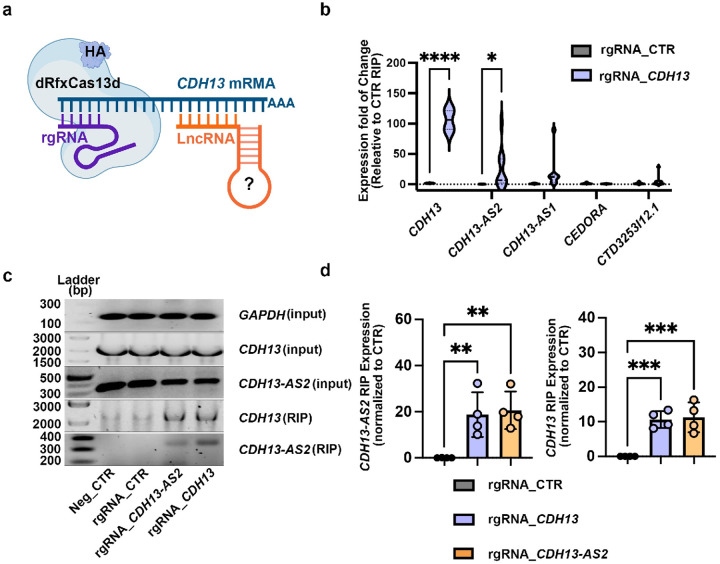
dRfCas13d-based RNA immunoprecipitation (RIP) identifies *CDH13-AS2* as a binder of *CDH13* mRNA. **a,** Scheme of dRfxCasI 3d-based RIP. **b,** rgRNA_*CDH13*/dRfxCas13d-based RIP identified, among the four IncRNAs, *CDH13-AS2* as the binder of *CDH13* mRNA in human ECs. qPCR was conducted using the RIP products and analysed using 2-way ANOVA multiple comparisons: *, P ≤ 0.05; ****, P ≤ 0.0001. N = 3. c,The representative electrophoresis of corresponding RIP experiments. Both rgRNA_*CDH13*/dRfxCas13d and rgRNA_*CDH13-AS2*/dRfxCas13d based RIP were able to pull down both *CDH13* and *CDH13-AS2*, confirming the binding of the two. Neg_CTR, negative control, d. The bar graphs are the statistics of the RIP experiments, one-way ANOVA multiple comparisons: *, P ≤ 0.05; **, P ≤ 0.01; ***, P ≤ 0.001. N = 4.

Given that lncRNAs often regulate gene expression *in cis*, we explored whether the four lncRNAs could affect *CDH13* expression. We first tested whether the four lncRNAs could directly bind to on the *CDH13* mRNA by conducting dRfxCas13d-based RNA-immunoprecipitation (RIP) in human umbilical vein endothelial cells (HUVEC). The dRfxCas13d construct was fused with an HA tag, allowing the anti-HA magnetic beads to pull down and enrich the binding RNAs^25^ (Figure. 3a). Five RNA-targeting guide RNAs (rgRNAs) of *CDH13* were used to pull down *CDH13* mRNA and its potential binders. The scrambled rgRNAs were used as controls (supplementary Table 6). Using the dRfxCas13d/rgRNA-*CDH13* system, we detected the significant binding of *CDH13-AS2* on *CDH13* mRNA, but not the other three lncRNAs (Figure. 3b). This might suggest the direct effects of *CDH13-AS2* on *CDH13*. To confirm this, we further investigated the interaction between *CDH13-AS2* and *CDH13* mRNA in HEK.293T cells, where neither transcript was expressed to minimize confounding factors. We first specifically activated the expression of *CDH13-AS2* and *CDH13* by CRISPR-mediated transcriptional activation (CRISPRa)^26^ (Figure. 5a). The sequence of dgRNAs was designed based on the promoter or enhancer of the gene target (supplementary Table 8). Five dgRNAs were used to activate the expression of *CDH13-AS2*, three and two dgRNAs, respectively, targeting the promoter (ENCODE ID, E1832741) and the enhancer (ENCODE ID, E1832742) of the lncRNA coding gene (Extended Data Figure 3a). We tested the effectiveness of the five dgRNAs individually to identify a highly potent dgRNA. All five dgRNAs significantly activated the expression of *CDH13-AS2* with high efficiency, and dg1_*CDH13-AS2* induced the strongest activation and was therefore used for the following experiments (Extended Data Figure 3c). To activate the expression of *CDH13*, we designed eight dgRNAs for this gene, dg 1 – 4 for the promoter (ENCODE ID, E1832187) and dg 4 – 8 for the enhancer (ENCODE ID, E1832188), all near the transcription start site (TSS) or exon 1 (Extended Data Figure 3b). Four dgRNAs, dg2, 4, 7, and 8, drove expression activation of *CDH13*, and dg4_*CDH13* showed the highest efficiency (Extended Data Figure 3d). In the HEK. 293T cells, we successfully induced high expression of both *CDH13-AS2* and *CDH13* using the corresponding CRISPRa (Figure 3c, Extended Data Figure 3c, 3d). In these cells, we transfected the plasmid system of dRfxCas13d/rgRNA-*CDH13*-based RIP, which was able to pull down the *CDH13-AS2* (Figure 3c). Likewise, we designed the dRfxCas13d/rgRNA-*CDH13-AS2* mediated RIP as previously, which successfully precipitated *CDH13* mRNA (Figure 3c and 3d, supplementary Table 6). These results were in line with our *in silico* experiments, in which we predicted the lncRNA-mRNA binding of *CDH13*, respectively, with the four lncRNAs using the LncRRIsearch webtool (supplementary Table 5). Among the four, we predicted *CDH13-AS2* as the only positive binder via reverse-complementary to the 3’UTR of *CDH13* mRNA. The local base-pairing interaction energy was −113.58 kcal/mol, representing the strongest interaction among the 100 anticipated bindings (Extended Data Figure 3e, 3f and supplementary Table 5). These experiments consolidated our findings on the interaction between *CDH13-AS2* and *CDH13*.

### *CDH13-AS2* positively regulates *CDH13* expression and EC functions

**Figure.4:**
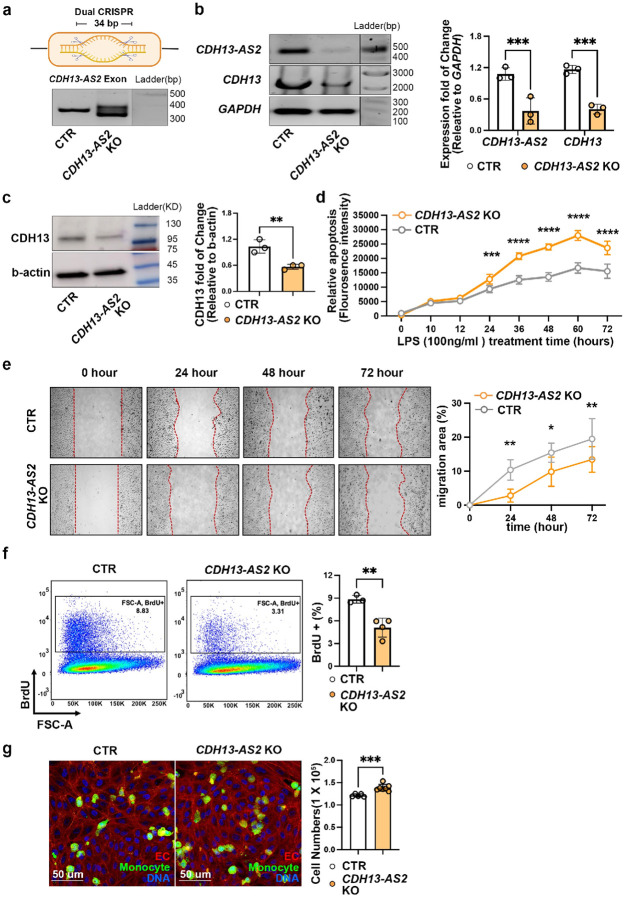
CRISPR-based knockout (KO) of *CDH13-AS2* in endothelial cells induces atherogenic phenotypes. **a,** Dual CRISPR-KO of *CDH13-AS2* in HUVECs perturbed the gene. **b,** Decreased the RNA levels of *CDH13-AS2* and *CDH13* in *CDH13-AS2* KO HUVECs compared to scrambled control cells (CTR) (2-way ANOVA multiple comparisons). **c,** Protein levels of CDH13 in *CDH13-AS2* KO vs CTR HUVECs (unpaired t-test). **d** through **g**, In HUVECs, KO of *CDH13-AS2* induced increased apoptosis under treatment of 100 ng/ml lipopolysaccharide (LPS) for 72 hours (N = 5, 2-way ANOVA multiple comparisons) **(d),** decreased migration in 72 hours (N = 5, 2-way ANOVA multiple comparisons) **(e),** reduced proliferation (unpaired t-test) **(f),** and increased monocyte adhesion (unpaired t-test) **(g)**.ln **(g)**, HUVEC cells were labeled by phalloidin (red), TPH1 monocytes by calcein (green), and DNA by DAPI (blue). *, P ≤ 0.05; **, P ≤ 0.01; ***, P ≤ 0.001; ****, P ≤ 0.0001.

To explore whether *CDH13-AS2* could affect *CDH13* expression and CAD-relevant cellular phenotypes, we knocked out *CDH13-AS2* (*CDH13-AS2*-KO) using the dual-CRISPR/Cas9 targeting strategy (Figure. 4a). The third generation of lentiviral system was used to deliver the sgRNAs and spCas9 transgenes (supplementary Table 7). The dual-CRISPR strategy excised a 34bp of the shared exon (ENSE00002602225) of *CDH13-AS2* major transcripts^27^ (Figure. 4a, Extended Data Figure 4a). Amplification PCR and qPCR on the KO lines showed a successful knockdown of *CDH13-AS2* expression (Figure. 4b). Likewise, we observed reduced *CDH13* mRNA and protein levels were in *CDH13-AS2*-KO HUVECs compared to control cells (Figure. 4b and 4c). Furthermore, we assessed functions related to EC fitness, including apoptosis, migration, proliferation, and immune cell adhesion, in the *CDH13-AS2*-KO HUVECs. To induce apoptosis, gene-edited cells were treated with 100 ng/ml LPS, and after the treatment, apoptosis-triggered fluorescence changes were detected every 10 or 12 hours until 72 hours. After 24 hours of treatment, *CDH13-AS2*-KO cells showed stronger apoptosis compared to control cells at each time point of measurement (Figure. 4d). To explore the migration of the gene-edited cells, we conducted the wound healing assay, and imaged cell migration every 24 hours until 72 hours. After wounding, *CDH13-AS2*-KO cells displayed slower migration into the wounded area at three time points (24, 48, and 72 hours), indicated by a lower percentage of cell coverage in the wounded area (Figure. 4e). The proliferation of *CDH13-AS2*-KO HUVECs was assayed by BrdU incorporation for 16 hours followed by flow cytometry analysis. The result showed decreased proliferation in the *CDH13-AS2*-KO cells (Figure. 4f). Increased EC apoptosis and reduced EC migration and proliferation could decelerate the healing of endovascular lesions and promote atherosclerosis. To probe the immune cell adhesion, we labeled THP1 monocytes with calcein, a live cell-permeant dye, and added the labeled cells onto *CDH13-AS2*-KO and control HUVEC cells. We observed increased monocyte adhesion on the *CDH13-AS2*-KO cells (Figure. 4g, Extended Data Figure 4b), which might contribute to increased immune cells in atherosclerosis plaques.

**Figure.5:**
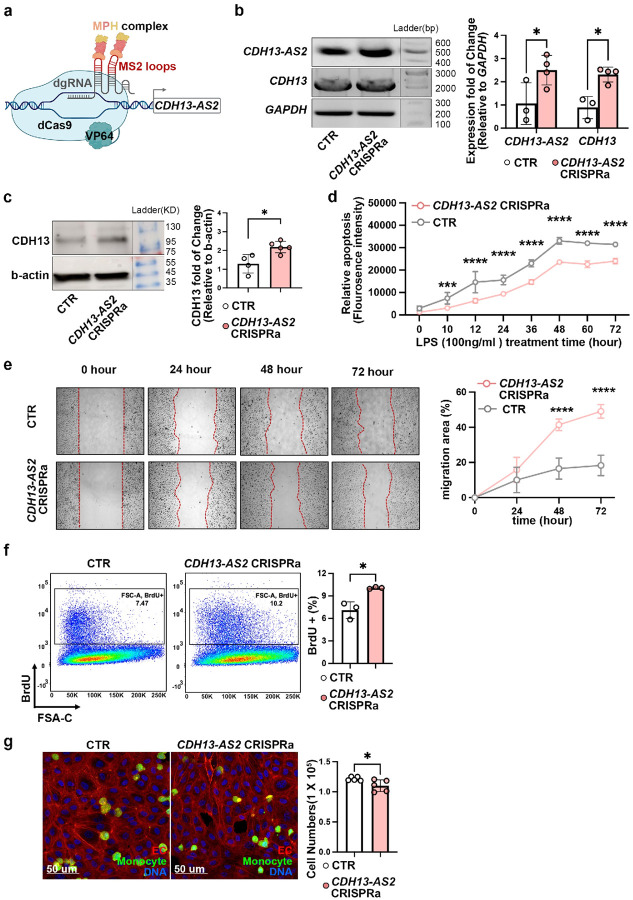
CRISPR-based transcriptional activation(CRISPRa) of *CDH13-AS2* in ECs drives atheroprotective phenotypes. **a,** Scheme of CRISPRa of *CDH13-AS2* using dgRNAs and enzymatically inactivated Cas9 fused with transcription activator VP64 (dCas9-VP64). The two hairpin aptamers (MS2) of dgRNA recruits the MCP:P65:HSF1 (MPH) complex to the enhancer/promoter of the gene. **b,** CRISPRa of *CDH13-AS2* in HUVECs increased itself and *CDH13* RNA expression (2-way ANOVA multiple comparisons). **c**, Protein levels of CDH13 in *CDH13-AS2* CRISPRa vs CTR HUVECs (unpaired t-test). **d,e,f,g,** In HUVECs, CRISPRa of *CDH13-AS2* suppressed HUVEC apoptosis under treatment of 100 ng/ml lipopolysaccharide (LPS) for 72 hours (N = 5, 2-way ANOVA multiple comparisons) **(d)**, increased migration in 72 hours (N = 5, 2-way ANOVA multiple comparisons) **(e)**, enhanced proliferation (unpaired t-test) **(f)**, and reduced monocyte adhesion (unpaired t-test) **(g)**.ln **(g)**, HUVEC cells were labeled by phalloidin (Red), TPH1 monocytes by calcein (green), and DNA by DAPI (blue). *, P ≤ 0.05; **, P ≤ 0.01; ***, P ≤ 0.001; ****, P ≤ 0.0001.

To test whether *CDH13-AS2* overexpression could lead to the opposite effect, we employed CRISPRa experiments in HUVECs as previously^26^ (Figure. 5a, Extended Data Figure 3c to 3f, Extended Data Figure 5a). Converse to the phenotypes in *CDH13-AS2*-KO HUVECs (Figure. 4), HUVECs with *CDH13-AS2*-CRISPRa had higher *CDH13* expression increased migration and proliferation, and decreased apoptosis and monocyte adhesion (Figure. 5b–5g, Extended Data Figure 5b). Thus, the enhanced level of *CDH13-AS2* increased *CDH13* expression and led to atheroprotective endothelial phenotypes, which might mitigate the risk of CAD.

### *CDH13-AS2* stabilizes *CDH13* mRNA

**Figure.6:**
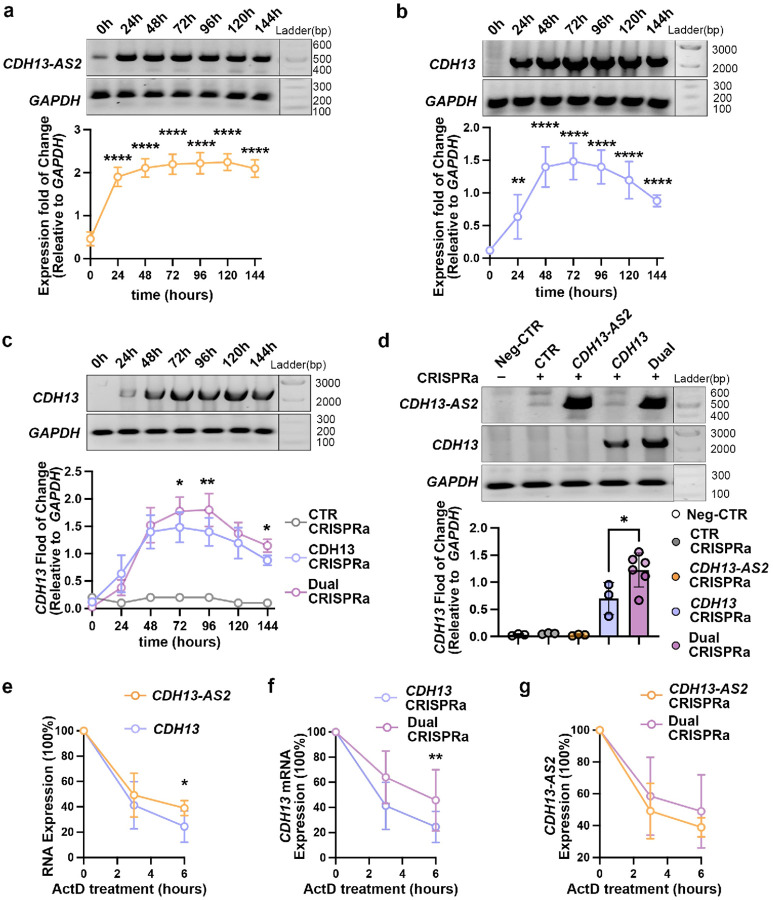
*CDH13-AS2* stabilizes *CDH13* mRNA. **a**, Expression increases of *CDH13-AS2* over time by dg1 *_CDH 1*3-/4S2/dCas9-based CRISPRa in HEK.293T cells (Extended Data Fig.5d) (N = 5, 2-way ANOVA multiple comparisons). **b**, Expression increases of *CDH13* overtime by dg4_CDH73/dCas9-based CRISPRa in HEK.293T cells (Extended Data Fig.5e), (N = 5, 2-way ANOVA multiple comparisons). **c**, Dual CRISPRa of *CDH13* and *CDH13-AS2* postponed the *CDH13* mRNA decay from 72 hours to 96 hours after transfection (N = 3, 2-way ANOVA multiple comparisons). **d**, At 144 hours, dual CRISPRa resulted in more *CDH13* mRNA than single *CDH13*-CRISPRa (ordinary one-way ANOVA multiple comparisons). **e**, Less RNA decay was observed for *CDH13-AS2* than *CDH13* mRNA, ~ 80% vs ~ 60% after actinomycin D (ActD) treatment (N = 7, 2-way ANOVA multiple comparisons). **f** and **g**, Comparing single vs dual CRISPRa, *CDH13-AS2* CRISPRa reduced *CDH13* mRNA decay by ~ 30%, while *CDH13* CRISPRa did not show a significant impact on *CDH13-AS2* expression (N = 7, 2-way ANOVA multiple comparisons). *, P ≤ 0.05; **, P ≤ 0.01; ***, P ≤ 0.001; ****, P ≤ 0.0001.

LncRNAs were shown to stabilize other RNAs^28, 29^, which likewise appeared to be feasible for *CDH13-AS2* and *CDH13* mRNA given their positive correlation. To test this hypothesis, we use a time-course CRISPRa experiment and an RNA stability assay. We conducted these experiments in HEK. 293T cell line again to minimize potential confounding effects (Extended Data Figure 3c to 3f). We conducted time-course experiments to observe RNA CRISPRa using plasmids encoding dg1_*CDH13-AS2*/dCas9. 24 hours after the transfection, we observed an approximately four-fold increase of *CDH13-AS2*, and the high expression level was maintained for 120 hours (Figure. 6a). At 144 hours, we observed a slight RNA decay of *CDH13-AS2* (Figure. 6a). The experiment showed good RNA stability of *CDH13-AS2*. Time course experiments of the dg4_*CDH13*/dCas9-based CRISPRa indicated that the activation of *CDH13* peaked at 72 hours after transfection (Figure. 6b). At 96 hours, we observed the mRNA decay of *CDH13*, which was 48 hours earlier than *CDH13-AS2* (Figure. 6a and 6b). However, additional CRISPRa of the *CDH13-AS2* in the same cells shifted the peak expression level of *CDH13* from 72 to 96 hours, postponing the mRNA decay by 24 hours (Figure. 6c). At the endpoint of the experiments (144 hours), cells with the additional activation of *CDH13-AS2* still showed higher *CDH13* expression compared to without (Figure. 6d). The results suggest that *CDH13-AS2* can stabilize the expression of *CDH13*. We further confirm this by testing whether *CDH13-AS2* could prevent *CDH13* mRNA decay after transcription blocking. In *CDH13* and *CDH13-AS2* CRISPRa experiments, after 48 hours of plasmid transfection, actinomycin D was used to block the gene transcription. After 6 hours of actinomycin D treatment, less RNA decay was observed for *CDH13-AS2* than *CDH13* mRNA, ~ 60% vs ~ 80% (Figure. 6e). Comparing the *CDH13* single vs *CDH13*&*CDH13-AS2* dual CRISPRa, *CDH13-AS2* overexpression was able to reduce *CDH13* mRNA decay by ~ 30% (Figure. 6f). However, *CDH13* overexpression did not affect *CDH13-AS2*, comparing the *CDH13-AS2* single vs *CDH13*&*CDH13-AS2* dual CRISPRa (Figure. 6g).Our data indicated *CDH13-AS2* as a stabilizer for *CDH13* mRNA.

### *CDH13-AS2* competes with miRNAs to stabilize *CDH13* mRNA

**Figure.7:**
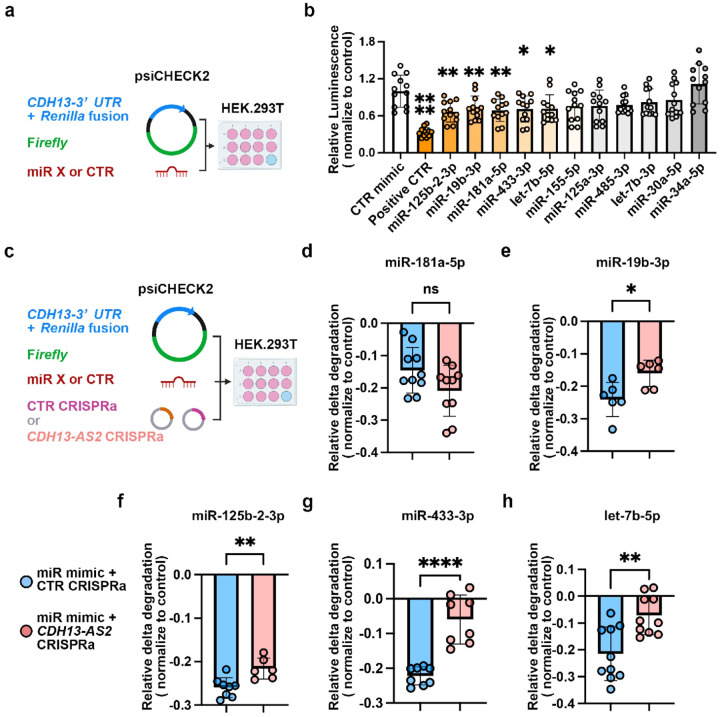
*CDH13-AS2* competes with four microRNAs for the binding on *CDH13 3’ UTR*. **a,** Experiment sketch of the RNA interference screen. **b,** RNA interference screen for miRNAs interacting with *CDH13 3’ UTR*. The name of the miRNA mimics is indicated. The negative control (CTR mimic) was a scramble miRNA mimic. The positive-CTR was a UTR sequence with four let-7b-5p binding sites in the psiCHECK vector (ordinary one-way ANOVA multiple comparisons). **c,** Experiment sketch of RNA interference rescue. **d to h,** Among the five positive miRNA candidates, except miR-181a-5p **(d),** four miRNA-mediated *CDH13 3’ UTR* decay was patrial rescued by *CDH13-AS2* CRISPRa, including miR-19b-3p **(e),** miR-125b-2-3p **(f),** miR-433-3p **(g),** and let-7b-5p **(h)** (unpaired t-test). *, P ≤ 0.05; **, P ≤ 0.01; ****, P ≤ 0.0001.

A ~1.6kb long 3’UTR was annotated for the *CDH13* coding gene by the Ensembl genome database (GRCh37.p13), and *CDH13-AS2* was predicted to bind to *CDH13* 3’UTR (supplementary Table 5, Extended Data Figure 3e and 3f, Extended Data Figure 6). Given that miRNAs can regulate gene expression by binding UTRs to trigger RNA decay^30^, we, therefore, tested whether there are miRNA(s) at the 3’UTR of *CDH13* mRNA. We first predicted miRNA binding on the *CDH13* 3’UTR using four databases, miRTarBase^31^, miRWalk^32^, scanMiRApp^33^, and TargetScan^34, 35^. The predicted miRNAs were further selected based on their expression level in ECs^36^ (supplementary Table 9). 63 predicted miRNAs with reads per million >20 were selected and further prioritized based on 1) being conserved between human and mouse, 2) experimental hints (RNA-seq), or 3) suggested by ≥ two prediction databases (supplementary Table 9). Finally, 11 miRNAs (supplementary Table 10), including miR-30a-5p, miR-181a-5p, miR-155–5p, miR-19b-3p, miR-125b-2–3p, miR-34a-5p, let-7b-5p, let-7b-3p, miR-125a-3p, miR-485–3p, and miR-433–3p were finalized for further investigation. The corresponding 11 miRNA mimics and a scrambled control mimic were purchased commercially (Qiagen) (supplementary Table 11). A 1323bp of 3’UTR covering all the predicted binding sites of the 11 miRNAs was synthesized and cloned into a dual luciferase plasmid (psiCHECK^™^−2|RNAi Assay, Promega) to generate the psiCHECK-UTR1323 construct. The scrambled control (CTR mimic) and the 11 miRNA mimics were respectively co-transfected with the psiCHECK-UTR1323 construct to functionally analyze potential miRNA binding sites on *CDH13* 3’UTR by an RNA interference assay. The assay is based on the Renilla/Firefly luminescence signals. Both luminescences were encoded on the psiCHECK backbone, the former was fused with the 3’UTR sequence, and the latter was on the same plasmid as the internal control (Figure. 7a). After co-transfection of the 11 miRNAs, respectively, with the psiCHECK-UTR1323 construct for 48 hours, both Renilla and Firefly luminescence were measured. In comparison to the CTR mimic, five miRNAs, miR-125b-2–3p, miR-19b-3p, miR-181a-5p, miR-433–3p, and let-7b-5p triggered Renilla luminescence reduction relative to the Firefly signal (Figure. 7b). demonstrating their effects on triggering RNA decay at *CDH13* 3’UTR. To probe whether *CDH13-AS2* could block the interaction of the five miRNAs with *CDH13* 3’UTR, we co-transfected control or *CDH13-AS2* CRISPRa plasmids, the psiCHECK-UTR1323 and correspondingly the five miRNAs in the HEK. 293T cells for 48 hours (Figure. 7c). Compared to the control, cells with *CDH13-AS2*_CRISPRa successfully restored the reduced luciferase activity caused by four of the five miRNAs, except for miR-181a-5p (Figure. 7d through 7h). The results demonstrated that *CDH13*-AS2 can inhibit miRNA-triggered *CDH13* mRNA degradation and therefore stabilize its expression. The lncRNA-miRNA-mRNA interplay might thus be harnessed to develop RNA-based therapies for CAD.

## Discussion

GWAS have reproducibly associated the *16q23.3* locus with the risk of CAD, but the underlying mechanisms remained unclear^1, 2, 18, 19^. Here, we identified *CDH13* and four lncRNAs as causal genes at the locus. In the UK Biobank, carriers of LoF alleles displayed increased risk for atherogenic traits. Colocalization analysis indicated *CDH13* contributing to CAD via its roles in the arterial wall(Figure 1a and 1b, Extended Data Figure 1a, supplement Table 1, 3 and 4). Moreover, advanced atherosclerotic plaques in the carotid artery displayed lower *CDH13* expression compared to early plaques(Figure 1d). Cdh13 protein levels gradually declined during the development of atherosclerosis in *Apoe*^*−/−*^ mice on a Western diet(Figure 1c). *Cdh13*^*−/−*^*/Apoe*^*−/−*^ mice on a Western diet developed larger atherosclerotic lesions compared to *Apoe*^*−/−*^ mice(Figure 1e). In line with our findings, Fujishima *et al*. reported enhanced neointima proliferation and atherosclerosisin *Cdh13*^*−/−*^/*Apoe*^*−/−*^ mice^37^. Thus, results from both humans and mice indicate that lower *CDH13* expression aggravates atherosclerosis or – *vice versa* – *CDH13* is atheroprotective.

Our GWAS-eQTL colocalization analysis also indicated four lncRNAs as candidate causal genes at the *16q23.3* locus(Figure 1b, supplement Table 1). We experimentally validated the *in-silico* prediction(supplement Table 5) that one of the lncRNAs, *CDH13-AS2*, binds to the *CDH13* 3’UTR(Figure 3b to 3d). CRISPR-based knockdown and activation of *CDH13-AS2* indicated a protective role of this antisense RNA(Figure 4 and 5), as it prevented *CDH13* mRNA decay and enhanced its beneficial effects in EC(Figure 6). We further demonstrated that four miRNAs were involved in the decay of *CDH13* mRNA. RNA interference and CRISPRa experiments revealed that the miRNA-triggered decay of *CDH13* mRNA was by ameliorated by *CDH13-AS2*(Figure 7).

Albeit more than 90% of GWAS signals map to non-coding regions only a few loci were found to act on CAD through ncRNA functions, such as MIAT at the *22q12.1* locus and ANRIL at the*9p21* locus^16, 38^, which is still the strongest CAD risk locus known so far^39^. At the *16q23.3* locus, we found that the lncRNA *CDH13-AS2* is one of the causal genes as it up-regulates the protein-coding gene *CDH13* in a *cis* manner by competing with the binding of miRNAs.

LncRNAs are commonly reported to bind to miRNAs, acting as sponges, to prevent mRNA degradation^40, 41^. A classic regulatory mechanism of miRNAs involves binding to the 3′ UTR of a mRNA, leading to its degradation or translational repression^12, 42–44^. Here we showed that *CDH13-AS2* competed with the binding of four miRNAs (miR-125b-2–3p, miR-19b-3p, miR-433–3p, and let-7b-5p)(Figure 7d to 7h). Similar interactions involving miRNAs have been reported for cancer and Alzheimer’s disease^40, 45^.

While current drugs for treatment of CAD uniformly address genes that increase CAD risk via lipid levels^1–3^, i.e. *PCSK9*^46^, *LPA*^47^, *ANGPTL3*^48^, and *APOC3*^49^, endogenous atheroprotective mechanisms are less well explored therapeutically. The potential of an atheroprotective milieu is illustrated by the internal mammary artery, which – even when exposed to high lipid levels or strong genetic disposition – resists plaque formation^50, 51^. In such a sense, our findings could be harnessed to design therapeutic strategies for CAD, going beyond lipid lowering, by increasing *CDH13* expression, thereby enhancing arterial resilience to the disease. From a therapeutic perspective, it may be challenging to interfere with the regulation of a widely expressed gene. In this respect, tissue or cell-specific regulation may be beneficial. To some extent, it is the case of *CDH13* given its expression in arteries limited to a few mesoderm-derived cell types (Figure. 2a–2d) and the CAD risk at the *16q23.3* locus is mediated by *CDH13* and lncRNAs exclusively in artery tissues. Indeed, endothelial cells demonstrated improved function upon activation via *CDH13-AS2*. The lncRNA-miRNA-mRNA interplay might inspire intravascular delivery of *CDH13* –miRNA target site blockers, antagomirs or anti-miRs to increase the expression of the gene, which protects coronary arteries from developing CAD. Target site blockers, anti-miR and alike, are already being explored as a therapeutic strategy for cardiovascular and cerebrovascular diseases as they can penetrate the arterial wall^15, 52–54^.

We are aware of the limitations of our study. First, although we were able to prioritize *CDH13* and the four lncRNAs as candidate causal genes for CAD by GWAS-eQTL colocalization analysis, no data were available to directly investigate the genetic link of the four miRNAs with CAD. Indeed, currently available population transcriptomes do not include sequences of such short RNAs. Second, our investigation provides mechanistic insights rather than specific therapeutics to increase *CDH13* expression, which may be challenging given that RNA delivery specific to ECs was shown to have low efficiency. However, RNA-based therapy, such as target site blockers or anti-mirs, showed good penetration into artery walls, which are already being explored for cardiovascular diseases^15, 52–54^. Additionally, further technological advancements are anticipated to enhance cell-specific RNA delivery. Third, *CDH13* was also expressed in VSMCs (Figure. 2a through 2f), which is worthy of an in-depth investigation but was not explored here. We rather focused on ECs that displayed high expression levels of *CDH13* as well as lncRNAs and miRNAs involved in its regulation.

Taken together, a lncRNA-miRNA-mRNA interplay affects the expression of *CDH13*, which resides as the only coding gene at a GWAS locus for CAD. Multiple lines of evidence indicate that the GWAS signal is mediated by the protective effects of *CDH13* on the arterial wall. The RNA interplay may be explored therapeutically by mimicking the effect of *CDH13-AS2* tointerfere with the miRNA-mediated degradation of *CDH13* mRNA at its long 3’UTR.

## Methods

### Colocalization analysis of the GWAS and eQTL signals

For colocalization analysis at the *16q23.3* locus, we used full-genome GWASs dataset of CAD, respectively, from CARDIoGRAMplusC4D and expression quantitative trait loci (eQTL) data of five tissue types of ~ 600 individuals from the STARNET (Stockholm-Tartu Atherosclerosis Reverse Networks Engineering Task) study^1, 55, 56^ (Figure.1c). The five tissue types included blood(n = 560), artery (atherosclerotic aortic root, n = 539 and free internal mammary artery, n = 553)), adipose tissue (subcutaneous, n = 534 and visceral abdominal, n = 534), skeletal muscle (n = 534), and liver (n = 546). Initially, we overlapped GWAS and eQTL summary statistics, utilizing a GWAS p-value threshold of 5e - 8 and an eQTL p-value cutoff of 0.01 to select SNPs for further testing. To estimate colocalization, we ran the coloc.abf function implemented in the coloc R package^57^ that uses an approximate Bayes factor to estimate the posterior probabilities between a given GWAS and eQTL signal (supplementary Table 1). Significant colocalizations were defined with PP4 ≥ 0.70, indicating a common causal variant between the GWAS and eQTL data.

### Differential expression analysis of *CDH13* using bulk RNA-seq data of patients

RNA sequencing data of human carotid artery plaques 57 early and 145 advanced plaques of the human carotid arteries were harvested during carotid artery endarterectomy (CEA) surgery, transported to the laboratory, and snap-frozen. CEA was performed due to advanced atherosclerotic lesion formation and stenosis in the carotid arteries. The patients’ characteristics are summarized in supplementary Table 2. The tissue handling,RNA extraction, and sequencing were as previously^58^. Bulk RNA sequencing and quality control (QC) were performed as described^23^. Raw read counts were normalized with the trimmed mean of M-values (TMM) and transformed with voom, resulting in log2-counts per million with associated precision weights, which were then used for differential expression analysis.

### Rare variant association analysis in the UK Biobank

The rare variant association analysis for *CDH13* was conducted using data from the UK Biobank (https://www.ukbiobank.ac.uk/) under the project 25214. We used whole-exome sequencing (WES) data of 470,000 participants from the UK Biobank (The final release of population-level exome OQFE variants)^59^. The UK Biobank annotation helper file was used to obtain the list of rare variants within the *CDH13* gene region. Variant annotation was derived from the snpEff tool using the Ensembl v85 gene definitions to determine their functional impact on transcripts and genes. Loss-of-function variants (LoF) included stop codon-introducing or splice site-disrupting SNPs, insertion/deletion variants predicted to disrupt a transcript’s reading frame, or larger deletions removing either the first exon or more than 50% of the protein-coding sequence of the transcript. We collected phenotypic data for 6 binary and 14 quantitative traits, including blood biomarkers, vascular health indicators, and immune cell parameters. The phenotypes were defined using the respective Field IDs, ICD-10 and ICD-9 codes obtained from the primary care, OPCS-4 diagnoses, and self-reported codes. When multiple measurements of the same phenotype were available for an individual, we utilized the measurements ofthe first visit. Age, sex, and 10 genotype principal components were gathered as covariates. The data was accessed through the UK Biobank Research Analysis Platform (RAP).

The association analysis was conducted on 500K WES data following the UKB tutorial ‘Burden Testing with regenie Using WES Annotation Files’ (https://dnanexus.gitbook.io/uk-biobank-rap/science-corner/using-regenie-to-generate-variant-masks#annotation-file). We used the genome-wide regression approach implemented in REGENIE^60^. Computationally efficient whole-genome regression for quantitative and binary traits. Nat Genet 53, 1097–1103. 10.1038/s41588-021-00870-7), available through the Swiss-Army-Knife tool library in RAP. Variants with an allele frequency below 1% were classified as rare. For association analysis, we used M1 variant masks, which LoF variants. To aggregate the effects of rare variants across the *CDH13* region, a burden test was applied. This approach consolidates the variants from our defined mask into a unified burden mask and evaluates it as a single genotype to produce association statistics. To assess associations with binary phenotypes, a logistic regression model was used, and for quantitative traits, linear regression. The output of the burden test included P-value, estimates of beta (effect size), and the standard error.

### Single-cell RNA sequencing (scRNA-seq) analysis

To characterize coronary artery expression of candidate genes at the *16q23.3* locus, we utilized the publicly available single-cell RNA sequencing (scRNA-seq) dataset GSE131778(Figure. 2a to 2f). This dataset includes single-cell transcriptomic data obtained from eight coronary artery samples derived from four patients who underwent heart transplantation. The samples were collected from the proximal-to-mid right coronary artery (RCA) after removing debris and selecting viable cells through fluorescence-activated cell sorting (FACS). The cells were processed using the 10X Genomics Chromium platform with 3’ chemistry reagents (version 2). The scRNA-seq analysis was performed following established protocols^24, 61–63^. Briefly, the scRNA-seq expression matrix was analyzed using the R package “Seurat.” Initially, gene expression levels were normalized using the “NormalizeData” function. Next, the “FindVariableFeatures” function was employed to identify the top 2,000 highly variable genes (HVGs) for further analysis. To reduce dimensionality, principal component analysis (PCA) was performed using the “RunPCA” function. Batch effects were corrected using the “Harmony” package, ensuring that the downstream analyses were not biased by inter-sample variability. To identify cell populations, the “FindNeighbors” function was applied to compute the k-nearest neighbors for each cell, followed by the “FindClusters” function to determine optimal clustering. The clustering resolution parameter was set to 0.5 to balance the granularity of cluster identification. To visualize the identified clusters, uniform manifold approximation and projection (UMAP) were utilized. Cluster annotation was conducted by referencing marker genes documented in the original study associated with this dataset^23^. Additionally, the “FeaturePlot” and “DotPlot” functions were utilized to specifically visualize the gene expression across various cell types within the dataset.

### Mouse studies

All mouse experiments were performed according to the regulations of German legislation on animal protection and were approved by the local animal care committee (District Government of Upper Bavaria, GZ: ROB-55.2-2532.Vet_02-18-177). The mice were bred and aged in the German Heart Centre Munich vivarium under standard conditions, following a 12-hour light/dark cycle with free access to food/water, maintaining temperature and humidity. Apolipoprotein E and Cadherin 13 double knockout (*Cdh13*^*−/−*^*/Apoe*^*−/−*^) mice were generated through crossbreeding *Cdh13*^*+/−*^ (purchased from The Jackson Laboratories, Bar Harbor, USA; *Cdh13tm1Brns/J*, subsequently termed *Cdh13*^*−/−*^) with *Apoe*^*tm1Unc*^ (purchased from The Jackson Laboratories, Bar Harbor, USA; subsequently termed *Apoe*^*−/−*^) mice over five generations. Experimental ages ranged from 12 weeks. Both males and females were included in the experiments, ensuring a balanced distribution of sex across treatment and genotype conditions. The mice were fed a Western Diet (MD88137 Adjusted Calories diet, 42% from fat, Harlan) for 4, 8, or 12 weeks to evaluate the progression of atherosclerosis. Both genotype groups were closely matched in terms of age. Experimental animals that died during rearing, suffered from other diseases, or failed to meet the scoring criteria (supplementary Table 12) were excluded. All remaining mice that met the scoring criteria were included for further experiments and statistical analysis.

### Histology

To evaluate aortic pathology, mice of both sexes in a balanced ratio were sacrificed at predetermined time points, as previously described (Extended Data Figure 1b and 1e). Euthanasia was performed using gaseous isoflurane, followed by transcardiac perfusion with 0.9% saline until complete replacement of circulating blood was achieved. The aorta and heart were exposed by carefully removing the surrounding tissues and subsequently fixed in 4% paraformaldehyde in PBS at 4 °C for 24 hours. The aortic roots were embedded in molds using an optimal cutting temperature (OCT) compound (Sakura Finetek, Tokyo, Japan) and snap-frozen on dry ice. Frozen tissue blocks were sectioned into 5 μm slices and mounted onto microscope slides for further analysis^64^.

### Enzyme-Linked Immunosorbent Assay

Mice arteries’ Cdh13 expression was assessed using a commercially available ELISA kit (abx518770, Abbexa) according to the manufacturer’s protocol. Aortic tissue, spanning from the aortic arch to the iliac artery, was excised, homogenized in 100 μL PBS using a tissue grinder, and centrifuged at maximum speed for 15 minutes at 4°C. The supernatant was collected for protein concentration analysis using the Pierce^™^ BCA Protein Assay Kit (Thermo Fisher Scientific) and subsequent ELISA assay. All procedures were carried out according to the manufacturer’s protocol. A total of 100 μL of each standard, test sample, and control (zero) were added to wells on a pre-coated plate, followed by a 1-hour incubation at 37°C. Subsequently, the plate was washed three times with 1X Wash Buffer, and 100 μL of Detection Reagent B working solution was added to each well. After a 30-minute incubation at 37°C, the solution was discarded, and the plate was washed five times. Next, 90 μL of TMB Substrate was added to each well, and the plate was resealed and incubated for 10–20 minutes at 37°C, protected from light. Lastly, 50 μL of Stop Solution was added to each well, and the absorbance was promptly measured at 450 nm.

### Oil Red O staining

The aortic root were imaged post-Oil Red O (ORO) staining to assess plaque burden. Tissues were briefly washed with PBS, then incubated at 37°C for 30 minutes in 3 mg/mL ORO solution (Sigma-Aldrich, O0625) in 60% isopropanol. Excess dye was removed with 60% isopropanol. Aortic arches were opened, pinned on a black pad, and imaged using a Stemi 2000-C microscope with an Axiocam ERc 5s camera and ZEN 2.3 Blue software. Plaque area at the aortic root was quantified by measuring at the maximum cross-sectional area. Aortic root sections were photographed after hematoxylin restaining of nuclei. ORO-positive areas were quantified using ImageJ, and lesion area percentages were calculated to evaluate atherosclerosis severity^64^.

### Cell cultures and passaging

HEK 293T cells (ATCC, USA) were cultured in high-glucose Dulbecco’s Modified Eagle Medium (DMEM; Gibco BRL, Grand Island, USA) supplemented with 10% fetal bovine serum (FBS; Gibco BRL) (supplementary Table 14). The primary human umbilical vein endothelial cells (HUVEC; PromoCell, pooled donors) were cultured in Endothelial Cell Growth Medium MV 2 (C-22022) supplemented with a complete supplement mix. All cells were maintained at 37°C in a humidified atmosphere with 5% CO_2_. The culture medium was refreshed every two days, and cells (HEK.293T, HUVEC) were passaged upon reaching 90% confluency.

Primary artery cells were handled using the DetachKit (PromoCell) following the manufacturer’s protocols (supplementary Table 14). The seeding density for HUVECs was maintained between 5,000 and 10,000 cells per cm^2^. THP-1 monocytes (ATCC, USA) were cultured in Roswell Park Memorial Institute (RPMI) 1640 medium supplemented with 10% fetal bovine serum (FBS; Gibco BRL) and 1% penicillin-streptomycin. Cells were kept at a density of 2 × 10^5^ to 1 × 10^6^ cells/mL to ensure optimal growth and viability. Cells were split every 2 – 3 days by diluting them with fresh culture medium. To induce differentiation of THP1 cells into macrophage-like cells, cells were treated with Phorbol 12-myristate 13-acetate (PMA) under a concentration of 100 ng/mL, 72 hours to trigger differentiation.

### CRISPR and DNA Cloning

Plasmids used in this study, including lentiCRISPRv2 (CRISPR knockout), lentiSAMv2 (CRISPR activation), pXR003:CasRx gRNA cloning backbone (dRfxCas13d-based RIP), and psiCHECK^™^−2 *CDH13* 3’ UTR (RNA interference assay), were commercially purchased, expanded in LB medium with antibiotics, and extracted using the PureYield^™^ Plasmid Mini/Max-prep System (Promega, A1223 /A2392). Linearization of lentiCRISPRv2 and lentiSAMv2 was performed using Esp3I (Thermo Scientific, FD0454), pXR003:CasRx by BbsI (FD1014), and psiCHECK^™^−2 by XhoI (FD 0694) and NotI (FD0593). Target fragments were purified via agarose gel electrophoresis and the QIAquick Gel Extraction Kit (28704). rgRNA, sgRNA, and dgRNA oligos were phosphorylated with T4 PNK (NEB M0201S) at 37°C for 30 minutes and respectively ligated with pXR003:CasRx, lentiCRISPRv2, and lentiSAMv2 using Quick Ligase (NEB, M2200S). The synthetic *CDH13* 3’ UTR (1323bp) was cloned into psiCHECK^™^−2 vector following the same procedures. Recombinant plasmids were transformed into Stbl3 bacteria, expanded, and screened on selective agar plates for positive clones following standard protocols.

### Cell transfection, lentivirus packaging, and cell infection

Plasmid encoding genes of interest or guide RNAs (supplementary Table 6,- 8 and 15) were transfected into cell lines using FuGENE^®^ HD (Promega, E5911), following the manufacturer’s protocol. Briefly, cells were seeded to reach 70–80% confluency on the day of transfection. Plasmid DNA, Opti-MEM^™^ I Reduced Serum Medium (Gibco), and FuGENE^®^ HD were mixed in a ratio corresponding to the seeding surface area. After a 30-minute incubation at room temperature, the transfection complex was gradually added to the cells in the well plate. Transfected cells were incubated in a humidified incubator at 37°C with 5% CO_2_. The time to harvest cells depends on different experiments.

To generate lentiviral particles, psPAX2 (Addgene-12260), pCMV-VSV-G (Addgene-8454), and guide RNA-expressing plasmids (supplementary Table 6 and 7) were co-transfected into HEK. 293T cells using the above transfection protocol. After 72 hours, the virus-containing supernatant was harvested, transferred to polypropylene storage tubes, and centrifuged at 2000 × g for 5 minutes to remove residual packaging cells. The clarified supernatant was filtered through a 0.45 μm PES filter, aliquoted, snap-frozen in liquid nitrogen, and stored at −80°C to preserve viral titer. For infection, HUCEV cells were incubated with the collected viral supernatant, diluted 1:1 with fresh culture medium, and supplemented with 10 μg/mL polybrene (1:1000 dilution) to enhance transduction efficiency. Cells were maintained in viral-containing medium for 48 hours before further processing.

### CRISPR/Cas9-based gene knockdown

To knock out *CDH13-AS2* using the CRISPR/Cas9 system, we utilized two sgRNAs and spCas9 to excise a 34bp of the shared exon (ENSE00002602225) (Figure. 4a). One lentiviral vector encoded by a sgRNA, spCas9, and a puromycin resistance cassette was used to pack the virus for gene targeting in cells. HUVEC cells were routinely passaged 48 hours after viral infection and subjected to positive selection after 7 days. Puromycin (1 ug/mL) selection was employed to eliminate negative cells after virus infection^27^.

### CRISPR/Cas9-based transcriptional activation (CRISPRa)

To increase the expression of RNAs, we employed CRISPR-mediated transcriptional activation (CRISPRa)^26^. The CRISPRa system includes two components, 1) the spCas9 lacking nuclease activity and fused with a transcription activator VP64 (dCas9-VP64) and 2) a short version of sgRNA (14–15 bp, dgRNA) tagged with hairpin aptamer (MS2) specifically recruiting transcriptional activation complex, MCP:P65:HSF1 (MPH) (Figure. 5a). The sequence of dgRNAs was designed based on the promoter or enhancer of the target gene (Extended Data Figure 3c to 3f). HUVEC cells were routinely passaged 48 hours after viral infection and subjected to positive selection after 7 days. Blasticidin (1 ug/mL) and Hygromycin (0.5 ug/mL) were employed to eliminate negative cells after virus infection.

### dRfxCas13d-based RNA immunoprecipitation

We performed RIP using the CRISPR system based on the RNA-targeting nuclease, Cas13. Several Cas13 orthologs have been found to recognize and target RNAs in mammalian cells, including LwaCas13a (139 kDa), PspCas13b (128 kDa), and RfxCas13d (112 kDa). We selected the enzymatically dead version of the smallest Cas13, dRfxCas13d (dCas13d)^25^, to explore the potential binding interaction among the four antisense lncRNAs and *CDH13*. The dCas13d construct was fused with a HA tag and a double-stranded RNA binding domain (dsRBD) stabilizing the dCas13d / rgRNA / target complex. The anti-HA magnetic beads were employed to pull down and enrich the binding RNAs and proteins. Five rgRNAs of *CDH13* were used to pull down *CDH13* mRNA and its potential binders (supplementary Table 6). The scrambled rgRNAs were used as the control(supplementary Table 6)^25^.

### RNA immunoprecipitation

HUVEC cells (1.5 × 10^7^) were washed with PBS and lysed in a buffer containing 50 mM Tris-HCl, 100 mM NaCl, 1% NP-40, 0.1% SDS, and 0.5% sodium deoxycholate at pH 7.4, supplemented with a Protease Inhibitor Cocktail Set III (EMD Millipore). Five percent of each lysate was reserved for input RNA preparation, while the remaining lysates were divided into two aliquots for immunoprecipitation. Immunoprecipitation was performed using 10 μg of either a specific antibody (CST, HA-Tag-C29F4, Rabbit mAb #3724) or control IgG (CST, Normal Rabbit IgG #2729), followed by incubation at 4°C for 8 hours. The immunoprecipitants were washed six times with a buffer containing 5 mM Tris-HCl, 150 mM NaCl, and 0.1% Triton X-100. Bound RNA fragments were isolated and purified using QIAzol Lysis Reagent (Qiagen, Cat. No. 79306) following the manufacturer’s protocols. The RNA underwent reverse transcription using SuperScript III with a random primer mix (Thermo Fisher, 12574026). The cDNA obtained was amplified using PCR for the target transcripts.

### Nucleic acid isolation

For DNA extraction from cultured cells, the DNeasy Blood & Tissue Kit (Qiagen,Cat. No. 69504) was used according to the manufacturer’s instructions. For the plasmid DNA from *E.coli*, the PureYield^™^ Plasmid Mini/Max-prep System, A1223 or A2392 was used following the manufacturer’s instructions.

Total RNA was extracted from cultured cells using QIAzol Lysis Reagent (Qiagen, Cat. No. 79306) following a modified Chomczynski and Sacchi protocol (1993). Cells were lysed with QIAzol and harvested using a cell scraper, then transferred to 1.5 mL microcentrifuge tubes. Following an incubation at room temperature, the lysates were vortexed, followed by the addition of 120 μL DNase-/RNase-free water and 100 μL chloroform. Samples were mixed by inversion, incubated at room temperature for another 10 minutes, and centrifuged at 13,000 rpm for 10 minutes at 4°C to separate phases. The aqueous phase was transferred to fresh tubes, combined with ice-cold isopropanol and 1.5 μL GlycoBlue^™^ coprecipitant (Thermo Fisher Scientific), vortexed, and incubated at −20°C for at least 30 minutes. RNA was pelleted by centrifugation at 14,000 rpm for 30 minutes at 4°C, washed twice with 75% ethanol, and centrifuged 10minutes at same conditions. Pellets were air-dried and resuspended in DNase-/RNase-free water.

DNA/RNA quality and concentration were determined using a NanoQuant Plate^™^ (Tecan) and an Infinite M200Pro spectrophotometer (Tecan), based on the 260/280 nm absorbance ratio. Resuspended RNA was either used immediately or stored at −80°C.

### Polymerase chain reaction (PCR)

Primers (17 – 30 bp) for PCR were designed using PrimerBank or Primer3 and synthesized by Eurofins Genomics (Ebersberg, Germany) (supplementary Table 16). Stock primers (100 μM) were diluted to 10 μM working solutions with DNase-/RNase-free water and stored at 4°C. cDNA was synthesized from mRNA using Maxima^™^ H Minus cDNA Synthesis MasterMix (Thermo Fisher Scientific) and stored at −20°C. Amplification PCR used Q5 2 x Master Mix (New England Biolabs) with primers (supplementary Table 16) and 750 ng of cDNA or 150 ng of genomic DNA. PCR products were resolved on 1 – 3% agarose gels depending on the product size, visualized under UV, and documented with Amersham ImageQuant 800 (Cytiva). qPCR was performed with PerfeCTa SYBR^®^ Green FastMix (Quantabio) on the ViiA 7 Real-Time PCR System (Applied Biosystems) using ~100 ng cDNA and primers (supplementary Table 16). Reactions were run in triplicate, normalized to housekeeping genes (ΔCt), and analyzed by the ΔΔCt method to calculate fold changes (2^-ΔΔCt) relative to controls.

### Protein isolation and Western blot analysis

All procedures were performed on ice unless stated otherwise. Cells were lysed in 1x RIPA buffer (NEB, 9806S) with HALT^™^ protease inhibitor (1:100, Thermo Fisher Scientific), incubated at 4°C for 30 minutes, and centrifuged at ~13,000 rpm for 30 minutes. Supernatants were collected, and protein concentrations were measured using the Pierce^™^ BCA Protein Assay Kit (Thermo Fisher Scientific) with absorbance at 562 nm (Infinite M200Pro spectrophotometer - Tecan). For Western blotting, 30 μg of protein was mixed with Laemmli buffer, boiled at 95°C, and resolved on 4 – 20% gradient gels (Bio-Rad) at 120 V. Proteins were transferred to PVDF membranes, blocked with 5% BSA, and incubated overnight with primary antibodies (supplementary Table 13). After washing, membranes were incubated with HRP-conjugated secondary antibodies (supplementary Table 13) and visualized using SuperSignal^™^ West Dura substrate on the Amersham ImageQuant 800 system (Cytiva).

### Wound healing migration assay

To perform the HUVEC wound healing migration assay, sterile forceps were used to position inserts (ibidi, No. 80369) into the plate wells, ensuring consistent alignment of the “wound field”. The cell suspension was prepared at a concentration of 0.5 × 10^6^ cells/mL in medium, and 300 μL of this suspension was carefully added to each well without disturbing the inserts. The plate was incubated overnight in a cell culture incubator to facilitate the formation of a confluent monolayer. To start the assay, inserts were gently removed using sterile forceps. The media was aspirated, and the wells were washed with fresh media to eliminate dead cells and debris. Following washing, medium containing 2.5 μg/mL of mitomycin C was added to inhibit cell proliferation, ensuring wound closure was driven by cell migration. The wells were observed under a light microscope, and additional washing was conducted if necessary. Cells were subsequently incubated, and wound closure was monitored at 24-, 48-, and 72-hours post-insert removal using light microscopy. ImageJ software was used to measure the percentage of closure or the rate of cell migration into the wound field.

### BrdU proliferation assay

For the cell proliferation assay, immunofluorescent staining of incorporated bromodeoxyuridine (BrdU) was performed using the BD Pharmingen^™^ APC BrdU Flow Kit (Catalog No. 557892), followed by the manufacturer’s protocol. To label cells with BrdU, 10 μL of a 1 mM BrdU solution was added per mL of culture medium, ensuring a cell density of no more than 1 × 10^6^ cells/mL to maintain normal cell cycling. After a 15-hour incubation, BrdU-plused cells were detached following washing and centrifugation at 200 g for 5 minutes. The cells were fixed and permeabilized on ice for 30 minutes using BD Cytofix/Cytoperm Buffer, followed by further permeabilization with BD Cytoperm Permeabilization Buffer Plus and refixed for 10 minutes. To detect incorporated BrdU, the cells were treated with DNase (300 μg/mL) and then incubated at 37 °C for 1 hour. After washing, the cells were resuspended in 50 μL of BD Perm/Wash Buffer containing diluted fluorescent anti-BrdU and incubated at 4°C for 20 minutes. The analysis of stained cells was conducted using a BD LSRFortessa flow cytometer (BD Biosciences) at a low flow rate to achieve optimal resolution. Gating was conducted for viable cells (forward scatter area [FSC-A] versus side scatter area [SSC-A]) and single cells (FSC-A versus forward scatter width [FSC-W], SSC-A versus side scatter width). Data analysis and statistical plotting were performed using FlowJo software v.9.9.6 (FlowJo LLC).

### Adhesion assay

HUVEC endothelial cells were seeded at 5,000 cells/cm^2^ on gelatin-coated 6-well or 12-well plates and cultured for 48 hours to form a confluent monolayer. THP1 monocytes were labeled with calcein dye (Invitrogen^™^, C1430) at a 1:1000 dilution, prepared at a concentration of 1.0 × 10^6^ cells/mL in serum-free RPMI-1640 medium, and incubated at 37°C for 60 minutes. After centrifugation, the supernatant was extracted, and the THP1 cells underwent two washes with serum-free RPMI-1640 before being resuspended at the same concentration for application onto HUVECs. The HUVEC endothelial cell medium was substituted with serum-free RPMI-1640, and 1 mL of the stained monocyte suspension was introduced into each well with the endothelial monolayer. Co-cultures were incubated for 180 minutes under standard conditions. After incubation, non-adherent leukocytes were removed by washing the wells three times with 2 mL of PBS. After the final wash, 500 μL of 1 X RIPA Lysis Buffer was added to each well for a 5-minute incubation at room temperature. After 14,000 × g centrifugation, 100 μL of lysate was then transferred to a 96-well fluorescence-compatible plate. Fluorescence was measured using the plate reader (Infinite M200Pro spectrophotometer - Tecan) at excitation/emission wavelengths of 480nm/520nm. A standard curve was generated based on a gradient of stained THP1 cells, and the number of adhered cells was calculated based on the fluorescence intensity, i-Control software was used for data collection (Tecan). For adhesion confocal microscopy images, endothelial cells were double-stained with Phalloidin (Invitrogen^™^, A12381) and DAPI, and images were captured after monocyte addition, incubation, and washing.

### Apoptosis assay

Apoptosis assay for gene-edited HUVECs was conducted using the RealTime-Glo^™^ Annexin V Apoptosis kit (Promega, JA1000) according to the manufacturer’s protocol. This live-cell, non-lytic, real-time assay detects phosphatidylserine (PS) exposure on the outer leaflet of the cell membrane during apoptosis. HUVEC cells were plated at a density of 5,000 cells/cm^2^ on gelatin-coated 96-well plates, incubated for 48 hours to establish a monolayer, and then treated with 100 ng/mL LPS. A 2X Detection Reagent was created by diluting each component 500-fold in the complete cell culture medium. An equal volume (100 μL) of the 2X Detection Reagent was added to each well. The assay was incubated for various time intervals, and apoptosis was assessed by measuring relative fluorescence units (RFU) using green fluorescence with excitation at 485 nm and emission at 525 – 530 nm (Infinite M200Pro spectrophotometer - Tecan). Fluorescence units (RFU) were assessed using M200Pro (Tecan) and i-Control software (Tecan).

### RNA stability assay

For the RNA stability assay, we treated cells with 10 μg/mL actinomycin D, a transcription inhibitor. The negative control (mock) was a medium with 10 μl/mL DMSO. After 48 hours of CRISPRa in cells, the culture medium was replaced with 10 μg/mL actinomycin D medium or mock medium and incubated at 37°C with 5% CO^2^. RNA samples were obtained after 0, 3, and 6 hours of treatment for RNA degradation comparison by qPCR.

### RNA interference assay

RNA interference assay was used for assessing potential microRNA binding on *CDH13* 3’UTR and conducted using the Dual-Luciferase^™^ Reporter Assay System (Promega, E1910) following the manufacturer’s instructions. The relevant 3’UTR region of *CDH13* consists of 1323 bp. The 1323bp sequence (supplementary Table 17) flanking the Xhol and Notl cutting sites was synthesized via GenScript Biotech (Netherlands) B.V. and inserted into the psiCHECK^™^−2 Vector backbone (Promega, C8021) via restriction enzyme cloning. For the assay, HEK.293T cells were seeded in 12-well plates and incubated at 37°C with 5% CO2. Upon reaching 70–80% confluency, cells were co-transfected in triplicate with 600 ng of the plasmid DNA construct and 50 nmol of microRNA. Following 48 hours, the cells were cooled on ice, rinsed once with 500 μL ice-cold 1× PBS, and then lysed with 50 μL 1× Passive Lysis Buffer (Promega, E1910). After centrifugation, 10 μL of the lysate was transferred to a white 96-well plate for a dual luciferase assay following the manufacturer’s protocol. Luciferase activities were assessed using M200Pro (Tecan) and i-control software (Tecan). The luciferase activity was quantified as the ratio of Renilla to Firefly luciferase activity and reported in arbitrary light units.

### Statistical analysis

The data of biological experiments were obtained from a minimum of three independent biological replicates. Quantitative results are expressed as the mean ± standard error of the mean (SEM). Statistical analyses were performed using GraphPad Prism, applying unpaired t-tests, one-way ANOVA with multiple comparisons, or two-way ANOVA for factorial analyses, as appropriate. Statistical significance was defined as *, P ≤ 0.05; **, P ≤ 0.01; ***, P ≤ 0.001; ****, P ≤ 0.0001. Description of sample size (number of LoF mutation carriers, non-carriers, total sample size) and statistical tests performed for genetic association analysis, can be found in the corresponding sections of the main text, Method details, and Supplementary Tables.

## Supplementary Material

Supplementary Files

This is a list of supplementary files associated with this preprint. Click to download.

- ExDFNCRRNAsat16q23Li.pdf
- supplementtableNCRRNAsat16q23Li.pdf

## Data Availability

This paper analyzes existing data of single-cell RNA-seq data from Gene Expression Omnibus (GEO), dataset GSE131778, GSE247238, and GSE247238. Original western blot images will be deposited at the required platform and publicly available as of the date of publication. Microscopy data reported in this paper will be shared by the lead contact upon request. All original code will be deposited at the required platform and will be publicly available as of the date of publication. Any additional information required to reanalyze the data reported in this paper will be available from the lead contact upon request.
